# TRANSFER‐I: Hospitalised Older Adults and Their Carers' Perspectives of the Transition Home: A Qualitative Study

**DOI:** 10.1111/hex.70343

**Published:** 2025-07-03

**Authors:** Kirsten J. Parker, Caleb Ferguson, Julee McDonagh, Richard Lindley, Louise D. Hickman

**Affiliations:** ^1^ Centre for Chronic and Complex Care, Blacktown Hospital Sydney Australia; ^2^ School of Nursing, Faculty of Science, Medicine & Health University of Wollongong Wollongong Australia; ^3^ Westmead Applied Research Centre University of Sydney Sydney Australia

**Keywords:** care transfer, frailty, integrated care, older adults, qualitative research, readmission, transitional care

## Abstract

**Background:**

Transitioning from hospital to home is a critical and fragmented process for acutely ill older adults and their carers. Despite widespread recognition of its importance, persistent gaps leave older adults vulnerable, highlighting the urgent need for safer transitions in care. Qualitative exploration into end‐user experiences of this transition can help to identify gaps in care and inform the development of targeted, person‐centred interventions.

**Aim:**

To explore the experiences of hospitalised older adults and their carers when they transition from hospital to home.

**Methods:**

Participants were purposively sampled during their contact with the rehabilitation and aged care service of a metropolitan hospital. Patients who had transitioned or were in the process of transitioning from hospital to home and informal carers to such patients were eligible. Verbatim transcripts were uploaded into NVivo and analysed using thematic analysis.

**Results:**

A total of 19 separate interviews were conducted, 12 patient and 7 carer interviews. The patients' mean age was 79 years (range 70–88 years), and carers' mean age was 74 years (range 58–85 years). Qualitative analysis developed three main themes during the transition from hospital to home, including (1) Impacting identity and the journey home: independence, frailty and functional ability; (2) Navigating inpatient care, communication and a harmonised transition; and (3) Pillars of support and the reality of social isolation.

**Conclusion:**

Complex challenges were highlighted for hospitalised older adults and their carers during transitions from hospital to home, reinforcing the urgent need for holistic, patient‐centred care. This study highlighted the compounding need to tailor discharge processes to individuals and calls for health services to embed patient‐centred discharge communication into service provision. These are essential steps towards enhancing the quality and safety of transitional care.

## Patient or Public Contribution

Patients and carers contributed to the design of this qualitative study through consultation with a consumer advisory group, where potential transitional care interventions were discussed. These discussions highlighted a need to further explore transitional care unmet needs, informing the development of this interview study with patients and carers. Participants' perspectives were central to the analysis and interpretation of findings. Participants received a simple one‐page summary of the findings, along with contact details for the research team to enable further comment or feedback.

## 1 Introduction

Older adults are among the most frequent users of acute hospital services, both for initial admission and subsequent readmissions [1]. As a response, ensuring safe and effective transitions in care has become a national health priority. Transitional care is defined as a set of actions to improve the communication and continuity of care between one healthcare setting to another or between levels of care [2]. Continuity of care activities ensure a safe transition home for at‐risk older adults, who have multiple chronic conditions, reduced functional capacity and higher adverse events [3]. Due to healthcare advancements, older adults are living longer, and as a result, there is a rise in people living with geriatric syndromes, such as frailty. Frailty is a condition marked by reduced physical strength and reserves, making individuals more vulnerable to illness and complications when faced with health challenges [4]. Shifts in population age distribution are expected to add significant pressure throughout the healthcare system, particularly acute hospital, long‐term care, community and primary services [5]. Predictions suggest the number of older adults aged 80 years and older will triple between 2020 and 2050 [6]. This projected rise calls for a re‐evaluation of existing care models and exploration into what constitutes effective transitional care for complex older adults. Inadequate transitional care practices have been linked to a range of adverse outcomes, increased iatrogenesis and a decline in safety at home [7]. Evidence suggests that well‐designed, targeted interventions can reduce readmissions and mortality among patients with complex care needs [8].

Given the complexity of care needs for hospitalised older adults living with frailty, it is critical to understand how contemporary transitional care processes are experienced from the perspectives of these populations. Previous qualitative research with older adults has demonstrated the importance of communication, interpersonal relationships, accessibility and navigation of services, community supports, patient education and information recall and self‐care in shaping transitional care experiences [9, 10, 11]. Transitional care is inherently multifaceted, involving coordinated efforts across multidisciplinary teams, resource availability and health system‐level planning [12]. However, if these processes do not align with what patients need or value, their effectiveness is limited. While clinicians play a critical role in preparing patients and carers for discharge, the success of these efforts depends on patient adherence to follow‐up plans, self‐management strategies and carer engagement [13, 14]. Given the diversity of healthcare systems globally, with significant variation in service models, resource allocation and policy priorities, it is essential to investigate how transitional care is experienced and delivered in local contexts [15].

The TRANSFER‐I study is based within a metropolitan health district that provides care to a highly diverse socio‐economic and multicultural population, with significant projected growth in the proportion of older adults within the community. Recent local data indicate that over 50% of individuals aged 65 years and over experience at least one all‐cause readmission in a 12‐month period [16]. Therefore, qualitative insights into complex older adults' and their carers' lived experiences of discharge from the hospital provide a foundation for evaluating the efficacy and appropriateness of existing transitional care interventions. Such an approach enables the identification of care discontinuities, systemic barriers and context‐specific areas for improvement. By spotlighting the voices of patients and carers, this study aims to inform the development of more responsive, person‐centred models of transitional care that can deliver meaningful health outcomes in an ageing and increasingly diverse population.

### 1.1 Aim

This study aims to explore the experiences of hospitalised older adults and their carers when they transition from acute hospital to home.

## 2 Materials and Methods

### 2.1 Study Design and Theoretical Framework

This study was approved by the Human Research Ethics Committee of Western Sydney Local Health District (2023/ETH00768) and site governance approval for Blacktown and Mount Druitt Hospitals (2023/STE01206) in July 2023. This descriptive exploratory qualitative study is reported following the COnsolidated criteria for REporting Qualitative research (COREQ) checklist by Tong et al. (2007) [17]. To explore the experiences of older hospitalised adults and their carers, 1‐1 semi‐structured mixed (telephone and in‐person) interviews were used. Interviews were analysed under contextualism theory [18], by emphasising understanding the surrounding environment, social interactions and culture that influence human behaviour and experiences. By situating data within its context, insights and understanding of the interplay between individuals and their environments are explored [19].

### 2.2 Participant Selection and Study Setting

Participants were purposively sampled during their admission from inpatient geriatric and rehabilitation wards at Blacktown and Mount Druitt Hospitals, based within the metropolitan Western Sydney Local Health District in New South Wales, Australia. These two metropolitan hospitals (with a total of 534 beds) manage thousands of admissions annually, and the health district serves a community with significant cultural, linguistic and socio‐economic diversity.

Patient and carer inclusion and exclusion criteria are described in Table 1. Any potential patients and carers were screened through Electronic Medical Records during the patient's admission. Eligible participants were approached in person by study personnel, provided information on the study alongside the Participant Information Sheet and invited to participate.

**Table 1:** Study inclusion and exclusion criteria for participants.

	Inclusion	Exclusion
Patient	Transitioning from hospital to home. Hospitalised older adults aged 65 years and above. Aboriginal and Torres Strait Islander peoples aged 45 years and above. English‐speaking.	Living in a residential aged care facility. Patients with a diagnosis of dementia or those without the capacity to consent.
Carer	Unpaid informal carer to a patient transitioning from hospital to home. Aged 18 years and older. English‐speaking.	Carers of a patient living in a residential aged care facility. Unable to consent.

### 2.3 Data Collection

Interviews were conducted either face‐to‐face (onsite at two metropolitan hospitals or at the participant's residential address) or via telephone, depending on the preference of the participant. All interviews were conducted separately, with either the patient or the carer. Interviewing patients and carers separately acknowledges that while they experience the same transition event, they do so with unique needs and concerns that merit independent exploration and analysis. This approach ensures that both viewpoints are considered and mitigates any power dynamics. With participants' permission, interviews were audio‐recorded. While family members of participants were occasionally present during interviews, they remained in the background and did not contribute to discussions. The research team developed interview guides from a review of relevant literature to explore patient and carer experiences, changes in patient needs after hospital admission, and suggestions for improving patient experience during the transition. Interviews were conducted by the first author K.P. The semi‐structured interview guides for patients (Appendix A) and carers (Appendix B) are included as appendices.

All participants were allocated a unique participant identifier. Patient demographic data was collected from Electronic Medical Records, including name, sex, postcode, Clinical Frailty Scale [20] (both pre‐hospital admission and on discharge) and the Charlson Comorbidity Index [21]. The carer demographic data collected was sex, age, relationship, cohabitation and postcode. Verbatim transcription was completed by either the first author K.P. or a professional transcription service (Pacific Transcription). Field notes collected during interviews were included in the analysis. Transcription occurred concurrently with data collection, allowing for content review and the development of key considerations. Interviews continued until data saturation was achieved [22, 23] and confirmed with the concept of information power, supported by a clear aim, purposive and specific sampling and quality of interview dialogue [24]. Each participant was offered to review their audio recording after the interview was completed, but all declined.

### 2.4 Data Analysis

Verbatim transcripts were read in their entirety and uploaded into NVivo (Version 13) [25], a qualitative software for coding and analysis. Qualitative reflexive thematic analysis was used, guided by Braun and Clarke's six‐step approach [26, 27]. Each transcript was individually coded by K.P., using an inductive coding approach. The research team then employed mind mapping techniques [28, 29] to systematically identify and organise codes into themes and to ensure that interpretations of data were consistent. K.P. and L.H. developed final themes from these discussions, which were approved by the extended research team to represent the experiences of participants during the transition from hospital to home.

### 2.5 Reflexivity and Rigour

The first author and interviewer, K.P., is a registered nurse and PhD candidate. Her supervisory team are the remaining authors, together holding extensive expertise in qualitative research, frailty science and health systems research. Trustworthiness of results was increased through credibility of data collection as the interviewer was an experienced health professional who did not work in the inpatient wards where participants had visited, thereby able to maintain objectivity and encourage both discussions of positive and negative experiences. All transcripts were checked for accuracy, and direct quotes from patients and carers were included to support interpretations of results. An audit trail was kept with process notes of coding and theme development through a consensus‐building process to increase confirmability [30, 31]. Descriptions of participants, study setting and research methods are reported to enhance transferability [32].

## 3 Results

**Figure 1:**
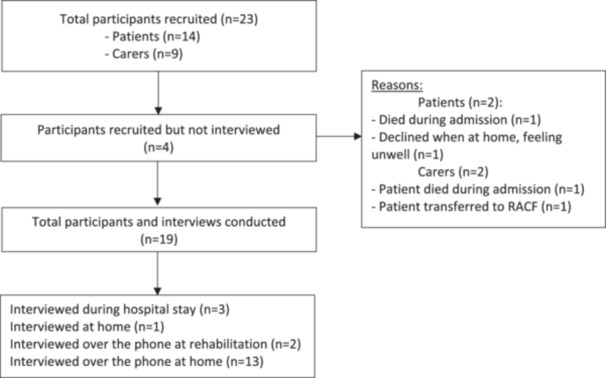
Interview participant recruitment flow chart for patients and carers.

A total of 19 separate interviews were conducted. 12 patient interviews (35% approach‐recruitment rate) and 7 carer interviews (56% approach‐recruitment rate). The participant flow chart with recruitment and withdrawals is outlined in Figure 1.

On average, interviews lasted 29 min, ranging between 13 and 64 min, in duration. Patients' mean age was 79 years (age range 70–88 years), and carers' mean age was 74 years (age range 58–85 years). At discharge, seven patients (58%) were classified as frail, with a Clinical Frailty Scale score of ≥ 5 (on a scale of 1–9, where higher scores indicate greater frailty) [33]. The mean Charlson Comorbidity Index for these patients was 5.75 ( ± 1.59), with higher scores suggesting a higher predicted risk of death within 1 year of hospitalisation [21]. Five (42%) of the patients who participated in an interview were also enrolled in the Western Sydney Clinical Frailty Registry [16]. Other participant characteristics and demographic information are presented for all participants in Table 2.

**Table 2:** Demographic information of interview participants.

Characteristic	Patient (*n* = 12)	Carer (*n* = 7)
Age (years)		
Mean	79	74
Range	70–88	58–85
Gender *n* (%)		
Female	6 (50%)	6 (86%)
Male	6 (50%)	1 (14%)
Coinhabiting *n* (%)		
Yes	5 (42%)	6 (86%)
No	7 (58%)	1 (14%)
Charlson Comorbidity Index		
Mean (SD)	5.75 (1.59)	—
Range	4–9	—
Clinical Frailty Scale		
Mean (SD) at preadmission	3.58 (1.11)	—
Mean (SD) at discharge	4.67 (1.10)	—
Range	2–6	—
Relationship to patient		
Wife	—	5 (72%)
Husband	—	1 (14%)
Daughter‐in‐law	—	1 (14%)

*Note:* All percentages were rounded to the nearest whole number.

Participant quotes are included to support qualitative themes, identified as either patients (P) or carers (C). Their sex (female [f] or male [m]) and age are also provided.

**Figure 2:**
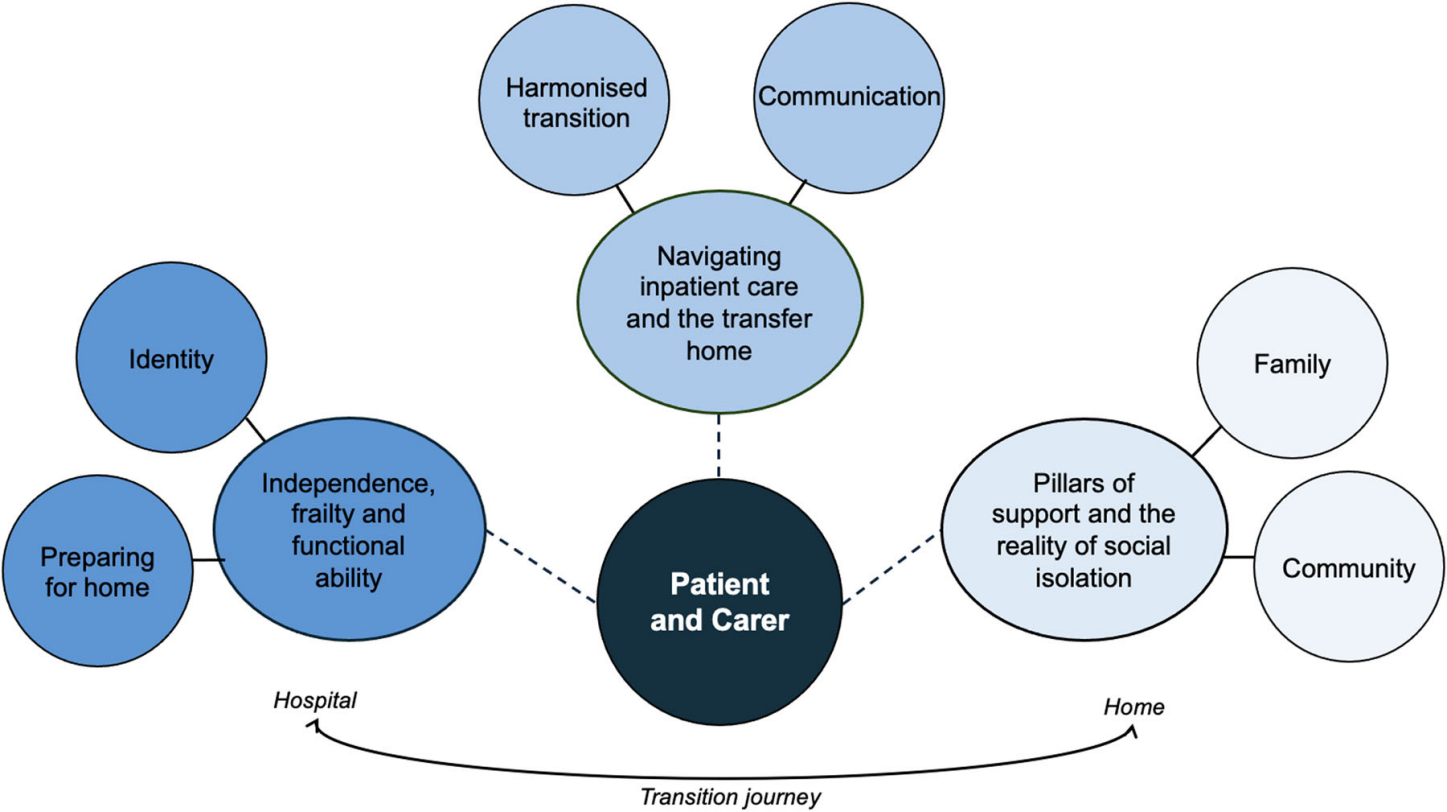
Visual representation of themes from the patient and carer transition from hospital to home.

Qualitative analysis of interviews developed three main themes from patient and carer transition from hospital to home, as demonstrated in Figure 2. These include:

1. Impacting identity and the journey home: independence, frailty and functional ability.
2. Navigating inpatient care, communication and a harmonised transition.
3. Pillars of support and the reality of social isolation.

### 3.1 Impacting Identity and the Journey Home: Independence, Frailty and Functional Ability

#### 3.1.1 Independence and Identity

Patients and carers highlighted independence as a crucial aspect of identity, shaping how individuals perceive themselves and their capabilities. If patients felt they were losing their independence, this shifted their identity perception. Patients were adamant about not wanting to lose this independence.

Well the biggest change for me as I was solely independent, I did everything for myself and suddenly I can do nothing for myself. I'm dependent on somebody to do everything now and I find that very hard’ … and … ‘I think you know, because to me, once I lose that functioning, life doesn't look too good.

P2, 87f

I can have all that, but I don't want that, I don't need. When it comes to the stage I need I'll get it. But to me it's taking my independence away.

P3, 70f

Loss on independence, I guess.

C7, 58f

The shock of being admitted to hospital and the unexpected nature of health decline raised questions for individuals about their ability to cope. Hospital admission created an unexpected and abrupt shift from independence to dependence for patients, and this was often emotionally and practically challenging.

The biggest change, obviously it was a surprise to be admitted, so yeah just not expecting any of this to occur therefore, I have to think carefully about what I do when I go home and how I look after myself…. The suddenness of it all.

P1, 71f

I'm a pretty independent person, I think. I hope I'm not stubborn, but a lot of people don't want any help, but you reach a stage you need help.

P9, 88f

This is something we haven't been through before. So, I wouldn't know what questions to ask…. So, this is all just new to us.

C5, 75f

#### 3.1.2 Functional Ability and Frailty Management

Navigating the changes of ageing and frailty for hospitalised older adults was highlighted through their daily experiences of managing chronic conditions. They emphasised concerns about physical limitations and the impact of the natural ageing process, collectively underlining the importance of addressing the diverse challenges patients face during transitions.

Bending down, because I'm afraid that if I do go down too quick or bend down I'm going to be on the floor again and I'm going to fall on my knees and my legs and I'll be back in hospital, and I don't want that to happen.

P7, 75m

Because I feel as if I'm deteriorating, and it's not what I want. I know I'm getting old, but not that old.

P8, 77m

Oh the back and the hips and that. And then I'll sit down on the lounge and I'll start getting the pains in the chest that goes through to me back … and I can't get up till it all goes.

P3, 70f

Carers highlighted the ongoing adjustments and medical interventions required to support individuals with chronic conditions. They emphasised the practical challenges of managing health decline and increasing frailty and this dynamic change in care needs over time.

And he had a bladder infection, that was the infection was starting to happen, these things were happening with him. So it's all part of old age. And he's been on antibiotics more times than he hasn't been in these months.

C1, 85f

There is other times we've—you know, we've got to keep him in pull‐ups now because he just loses his bladder every now and then and that sort of thing. Which he never did before.

C2, 72f

Patients and carers revealed concerns regarding the quality and accessibility of hospital meals, highlighting a shared sentiment that inadequate nutrition hinders recovery and the transition home. Concepts of nourishment neglect and how a decrease in weight can contribute to poor health outcomes were discussed.

What they gave him, it was like cat spew. It was ridiculous, no wonder he lost weight.

C2, 72f

It's a very important part of the hospitalisation. If they can't eat it, they're not going to get any better.

C1, 85f

#### 3.1.3 Preparing for Home: Importance of Services and Creating a Safe Environment

Patients and carers discussed the preparation required to safely return home from the hospital, highlighting the necessity of accepting assistance and additional care to ensure a smooth transition. Patients acknowledged the need for domestic and personal care support at home and often relied on the hospital to help organise and enact this support, including assistance with shopping, cleaning, gardening and personal care.

I knocked it [support] back, knocked it back, knocked it back. But after this last experience I had in the hospital I've decided to say yes. So I think I'll start saying yes to a few things now that can help.

P7, 75m

Well, at this stage, as soon as possible to be honest with you, because I need some help at home, and to get the help I don't know. That should have happened while I was in hospital, but unfortunately it didn't happen.

P5, 75m

During inpatient stays, assessments of a patient's functional ability and mobility, including activities of daily living and walking, were imperative for determining their ability to cope at home. Discharge preparations involved evaluating their capacity to manage these tasks and identifying potential challenges to predict safety at home. Patients emphasised the importance of the multidisciplinary team discussing the home environment to ensure it met their specific needs.

Because they discussed the home modifications which I have, and that is—I have railings in the bathroom, I have a handheld shower. I have railings at the front and back of the house for the steps. As I said, I have a walker, which My Aged Care provided.

P8, 77m

They asked all those questions, what you got at home. Then they said you need—well, I had a walker at home, but that I needed a shower chair. Because the hardest part was if you're in pain to sit on the toilet, they're too low.

P9, 88f

A recurring issue in interviews was the importance of services in the community. Patients described a shift in their willingness to accept help after difficult experiences. This change in attitude marked a significant step towards embracing the support needed to maintain independence and well‐being. While participants acknowledged the need for services, they often faced barriers to navigation and delays due to waiting times. This inadequacy of support impacted security and safety at home.

I do with, with that added care, that was the key that she had to have the added care at home.

C7, 58f

She led me through the minefield of aged caring. Because it's a minefield. Even though I had a brain, it's still a minefield.

C1, 85f

They said they thought I should—I'm on a Level 3 package from My Aged Care, and they felt I should be on a Level 4. I think the welfare officer there was going to put in a recommendation. A recommendation has been put in before.

P8, 77m

Then they're waiting for the funds, that's the thing. I have to wait for funding.

P6, 81f

### 3.2 Navigating Inpatient Care, Communication and a Harmonised Transition

#### 3.2.1 Treatment Transparency, Importance of Communication and Clinical Expertise

Hospitalised older adults valued understanding their medical treatments and management. Participants had an expectation to be involved in shared decision‐making about their treatment options. Additionally, there was a shared frustration with the lack of transparency regarding test results, which left patients feeling uncertain about their health status. Carers highlighted the importance of receiving verbal and written information directly, especially when caring for patients who have cognitive impairments, to ensure accurate understanding and decision‐making.

I mean really when it's all said and done it's just knowing diagnosis and medical treatment and putting that in place and then seeing the particular specialist and related specialists as outpatients, the medical side of it all.

P1, 71f

Well the biggest change that was in the hospital is not being told what's been going on … they have blood tests and all this tests and all this, they don't tell you what's going on. And I'm still in the dark with some of the tests.

P10, 80f

Yeah, the doctors, I wanted answers and I said, I don't seem to be here when youse come and I'm here most of the day. I said, but don't talk to John about it because he's got dementia and he forgets.

C2, 72f

As participants highlighted, managing medications and understanding their effects is a critical aspect of patient care. Carers and patients describe the significant impact of medication adjustments and polypharmacy on health outcomes. Medication reviews by skilled clinicians were important for their overall health and well‐being.

So he was on a lot of medication which they've actually cut out nearly all of it. They've told me not to give him hardly any of the medication he was on and he's actually got better since that.

C4, 76f

Once the tablets have been changed and been controlled, I became good.

P5, 75m

Effective management of inpatient needs and clear and concise communication about these needs were imperative. Participants highlighted the essential role of communication and collaboration in providing effective care. The need for consistent information, active listening and the involvement of both patients and their families became apparent. Shared decision‐making emerged as particularly important to make informed choices about their treatment and management, for example medication changes and plan for home including additional services and home modifications.

So everyone has to be involved in the care plan … so yeah because when everyone knows what's going on you don't get any misinformation.

P1, 71f

I think that that would be advantageous and I think that it's sort of like for everybody, for Mum to hear everybody on the same page…. But I think that would have been beneficial for everybody to be hearing the same thing at the same time and have that plan.

C7, 58f

Oh, yeah, anything that involved me and my future, of course I have to be involved in it, and not only myself because my daughter as well is trying to drive the whole thing together for me.

P5, 75m

Yeah, bloody oath, mate. It's about me. So, hey, we'll all sit down and we'll talk about me.

P3, 70f

However, some patients felt the communication from the medical and multidisciplinary team was poor or insufficient, impacting their hospital admission and transition home. Poor communication often left patients feeling dismissed and unheard.

Right there's no communicating. They might tell my son or my daughter more, but they're not communicating to the patient.

P10, female

There was no communication. Not even to the extent where I kept saying, I've got a sore back. There was no communication … dismissive is a very good word.

P12, 77m

They [nurses and doctors] gotta listen, don't judge people. You don't even know what they've been through so stop judging people. And because you're Aboriginal, you get treated like you're a freaking junkie and an alcoholic and everything else.

P3, 70f

Participant experiences varied, and the perceived workload of clinicians and staff emerged as a key factor impacting a patient's stay in the hospital. Participants described a busy hospital environment compounded by staff shortages, empathising with staff but, as a result, felt some discomfort or neglect.

I'm not criticising anyone. I know the staff are flat chat there, they said they don't even have lunch or anything.

C6, 83m

I had to wait till they, it was 3 days before I could have a shower, but nobody could help me in and out of the shower for 3 days. And I reckon I stunk, but they reckon I didn't stink but I felt uncomfortable.

P10, 80f

While this is the case, there was also a perceived sense of trust in the multidisciplinary healthcare team. This included positive interactions with staff and a sense of security in the expertise and dedication of clinicians. Namely, a sense of satisfaction with the care received.

I have trust in the medical team to look after me.

P4, 82m

I think the hospital was very supportive, especially the staff. I'm very thankful for the staff, the nurses, they're so helpful.

P6, 81f

Well, I think the doctor here has been very open and friendly and told me everything I needed to know or I feel they've told me everything I needed to know. So I'm pretty satisfied in that result.

P2, 87f

#### 3.2.2 Ensuring a Seamless Transition and Post‐Discharge Support

The discharge process and planning from hospital to home were critical. Patients and carers report mixed experiences regarding the timing and communication of discharge plans. Frequently, discharge notices were given with minimal warning.

They said that they think I'm ready to be discharged, I think I was—I just—I don't know how I felt. I felt like I wanted to stay more.

P6, 81f

They were pretty quick to get the beds free and I mean I could walk, I was sitting there watching TV. Then they already started tidying up, so I felt there was an urgency.

P9, 88f

Well, they just said what they had done for me in the hospital was as much as they could do at that time.

P11, 71m

Participants' experience of the communication around discharge processes and the plan for home was varied, as some stated there was insufficient discharge information, which left them feeling unprepared for discharge. Some participants had received discharge information, but it was not discussed with them in layperson language, or they were only given their discharge paperwork without detailed instructions.

Just a lack of information that I had for when I get home…. They couldn't stop and talk to me for so long and they're only there to assist you getting better to get home and that's about it.

P7, 75m

But it was actually none. It was actually none to be quite truthful. I was told I was going home and did I have anybody home here, I said yes.’ And ‘All they did is they just brought it up over and said that was my copy. That's where I had to go to the doctor. I've gotta make this appointment and that appointment. And that was it.

P10, 80f

Slightly confusing, yeah. A lot of medical stuff which I didn't understand, but it was not too bad …’ and ‘I didn't realise that my doctor had to make the appointment [scan of the brain]. He wasn't sure about that, and then he finally found it in the discharge letter. Gave me a referral for the CT scan for the—one of the brain, they're going to burn the brain out … [laughs] … if they find it.

P8, 77m

Alternatively, carers described being well‐informed about follow‐up care, including future doctor visits and tests. They depicted clear communication and thorough planning by hospital staff and clinicians. Additionally, carers received clear discharge documentation to ensure continuity of care to the community and various specialist follow‐ups.

They've informed us all about that and told us we'll have to go to the doctor and that it'll be up to the doctor how often he wants to see her with the warfarin and all of that.

C6, 83m

He's ordered some follow up tests as well, and for us to contact his heart doctor and all that sort of stuff. So we've got all that in motion. So yeah, yeah, so the staff that knew he was going to be discharged touched base and said we would get these appointments, which they've all rung to say this is what we'd like to do.

C3, 69f

Absolutely they did. So I had a pretty big note of all the things that they wanted Mum's GP to address, you know, to get certain tests done and to follow up on so, yes, definitely there was a conversation about that and that was with the doctors that were looking after Mum.

C7, 58f

The critical role of discharge communication and General Practitioner (GP) follow‐up in managing health post‐discharge was clear. The proactive efforts required to coordinate care, the challenges of navigating medical appointments, and the crucial role of GP's in ensuring continuity of care were all identified.

Yes, I did that myself, you know, I went to the doctor's, got the referral and all that. And then I booked it, then I rang up and booked the, booked for the specialist and that to go and see them.

P10, 80f

It was mainly the list of tablets that I had to get when I was trying to get hold of her. That was my main aim, to get the tablets on scripts, if they can be put on script, so I could get them to start taking them.

P7, 75m

Participants were asked to think of health service innovation and how the transition from hospital to home could be improved. Patients highlighted the important role of an advocate to assist with navigation (e.g., nurse navigator), to provide reassurance and support. They described how timely follow‐up from the hospital post‐discharge could help ensure a smooth transition home.

They become an advocate for that person and I suppose where some people just don't know the lingo or how things are done and that person becomes a base and they have to simplify all the details and help them through it and contact all these different services and make sure they understand the situation they are dealing with.

P1, 71f

It depends on the person. Some people might like one or two, with me just to follow up. You know, you got home alright? Blah, blah blah. Would you like me to call you back? And I would say no. You know, fair enough. And some of them, you know, they might not have anybody and just to follow up to see how they are would be terrific.

P10, 80f

I think they should follow up within a day or two. Second day, probably. How are you getting on? Do you need anything? Do you need home nursing?

P12, 77m

### 3.3 Pillars of Support and the Reality of Social Isolation

#### 3.3.1 Carers and Family Are Pillars of Support

The role of carers and family support was crucial in the lives of hospitalised older adults. Participants described a deep reliance on their family members for support in the form of daily activities, medical appointments, organisation of healthcare needs, socialisation and companionship.

You know, even going from one doctor to another, I rely a lot on my daughter to help take me out.’ And … ‘Without them I wouldn't have been supported.

P5, 75m

No. I always give her a hand. She could possibly do it herself, but I just like to be there with her to make sure she doesn't fall or anything.

C6, 83m

Patients described how the connection with family provided a sense of security during their journey home. The advantage of having family nearby and the gratitude for this support were apparent. Family members provided both emotional and physical support and were considered key to a successful transition.

‘I was glad that my daughter stayed with me for 2 weeks until I was fully comfortable to be on my own.’

P6, 81f

I've heard it before that they say, but I'm lucky, I got family that live close by. People who haven't got family, I wonder how they cope.

P9, 88f

During interviews, carers shared their own challenges, often feeling the weight of responsibility for their family members. Formal support and services frequently did not meet the needs of these older adults, leading family carers to fill the gap. The increased demands of caring and the concepts of carer stress, strain, burden and fatigue became apparent during interviews.

They do a bit of cleaning, vacuum and wash the floors and make the bed. That's what I do get after the shower but the rest—I mean that's only 3 days a week. There are 7 days in a week, I do the rest. I still do the cooking and all that and all the running around shopping, and doing all the bills and everything.

C2, 72f

I worry doing shopping and leaving him alone for an hour, a couple of hours. I need to go to the shops too, I need things but can't go and he hasn't been up to going with me. He won't go with me half the time. So that's my main worry.

C2, 72f

But then there was times I had to shower him, and had to get him into the shower and use that shower. And I was finding that difficult. I was starting to find that difficult.

C1, 85f

#### 3.3.2 Social Isolation and the Struggle at Home

Transitioning from hospital to home can be complicated for older adults without a carer or family support. Being alone at home exacerbated feelings of vulnerability and helplessness. Without the support of carers, family or formal services, older adults found it challenging to manage daily tasks, maintain their health and well‐being, and stay connected with their community. Participants reflected on the struggle of managing at home compared to the 24/7 care provided in the hospital.

It is really hard. Between you and I, I'm struggling at the moment’ and … ‘all I thought was how am I going to get home and how I'm going to survive at home, at this stage, but now I'm at home I need all the help I can get.

P5, 75m

Yeah, no, I think Mum found it a lot harder than what she thought she would.

C7, 58f

Put it this way, if my wife hadn't been able to come over, I would've been in deep trouble because I would've had to do everything myself. Go to the doctor, go shopping, go—because I had nothing, no one.

P12, 77m

Patients and carers identified other struggles faced in the community, including transport and financial costs. This highlighted the physical and logistical difficulties faced by individuals as they navigate the complexities of ageing at home, outside of institutional care.

Taxi, I've been doing. I tried the bus, and I couldn't get up off the seat. One of the other passengers helped me. I don't know how I'd do on getting on and off a train, but mind you, I've got to get to the train first of all …

P8, 77m

Yeah, but what does it cost us if we have that support?

C5, 75f

Yeah, I've gotta pay her [Occupational Therapist] $300.00 for one visit. It's the only way to get my stuff.

P3, 70f

While the demands of caring, social isolation and managing at home are significant, participants discussed how they would rather endure at home than transition into a residential aged care facility. Participants spoke about their negative perceptions of these facilities, as emotional and practical factors influenced the decision. Participants had concerns that this transition into an institution would considerably affect their quality of life, preferring to continue battling the challenges at home.

But we went and looked at two places and looked at it and I thought—no he probably wouldn't survive cause I'm looking after him anyhow and I'm giving him the home cooked things and familiarity and stuff and I wouldn't have been happy there. I'd have died very quickly, you know. It's just not the same.

C1, 85f

I wanted him home because he's not going in no nursing home.

C2, 72f

I certainly don't wanna go into a home or even one of those unit things. So I don't want…. I'd rather battle on alone somehow.

P2, 87f

## 4 Discussion

This study provided a greater understanding of the experiences of hospitalised older adults and their carers transitioning home through three main themes identified: (1) Impacting identity and the journey home: independence, frailty and functional ability; (2) Navigating inpatient care, communication and a harmonised transition; and (3) Pillars of support and the reality of social isolation. The TRANSFER‐I findings highlight persistent issues that have compounded over the past two decades [34] due to the increasing complexity of healthcare systems and an ageing population. Cultural factors, social isolation and financial limitations are common aspects of everyday interactions, yet despite these demographic variables, core issues remain unchanged—effective communication, appropriate information and discharge planning, and access and availability to support are essential for improving transitional care.

The TRANSFER‐I first theme, *‘impacting identity and the journey home: independence, frailty, and functional ability’,* highlights how declines in mobility and function significantly disrupt older adults' identity and self‐perception. Independence is closely linked to psychological well‐being and confidence in living alone and has long been recognised as a key priority for community‐dwelling older adults [11, 35, 36]. Research confirms the impact of physical limitations when discharging home from hospital, alongside insufficient services and assistance, on patients' ability to cope at home and their incidence of readmissions [37, 38]. Despite these obstacles, many strive to remain at home and avoid institutionalised care, relying on clinicians for comprehensive assessments and care plans to preserve their autonomy [39].

Secondly, ‘*navigating inpatient care, communication and a harmonised transition’* identified gaps in patient‐centred communication during hospitalisation and transfer home, with perceived staffing shortages and the known pressures to reduce hospital length of stay creating barriers to good communication. Enhancing shared decision‐making serves as a mechanism for improved patient activation and patient engagement, which leads to better health outcomes, as confirmed by previous literature [40, 41]. While some participants valued follow‐up support after discharge, others perceived it as relevant for older adults with more complex needs only. This follow‐up support is not a novel intervention and has been incorporated into various transitional care models with mixed effects on patient outcomes [42, 43]. Previous research has shown effective interventions share common features: they are multicomponent, include both inpatient and community contacts, and utilise skilled transitional care personnel [44].

The third theme ‘*pillars of support and the reality of social isolation’* highlights the critical yet often under‐recognised role of family and carers in bridging gaps in transitional care. The TRANSFER‐I study and previous research highlight the heavy reliance on informal carers for successful discharge [45, 46]. While formal services such as domestic assistance and home care are essential, they often fall short of the level of care that complex older adults require to safely manage at home [11]. These challenges are compounded by systemic fragmentation, long wait times and limited access to services [47]. Although government‐funded formal care services exist, they do not reach all older adults living at home, leaving family members to fill the gap [48, 49]. For those living alone, this gap is more pronounced. These participants described the challenges of managing daily life post‐hospitalisation and found the transition home more difficult than expected, with evidence linking social isolation to higher mortality risk [50]. While informal carers bridge this gap and improve survival at home, the practical limitations of informal care cannot be ignored, such as carer burn‐out, limited resources or the absence of support, which can leave older adults vulnerable [51].

### 4.1 Implications and Future Recommendations

For clinical practice, findings highlight the need for healthcare systems to adopt more holistic discharge approaches that extend beyond the hospital walls, recognising the complex interplay between medical needs, social determinants and system‐level factors in facilitating successful transitions for older adults. Prioritisation of patient‐centred discharge communication that promotes transparency and patient engagement is necessary. Personalised discharge summaries in simple language can significantly benefit vulnerable older adults, enhancing their understanding of post‐discharge care. Our findings strongly suggest exploring continuity of care models, particularly examining the efficacy of nurse navigators in discharge communication and post‐discharge follow‐up. Studies should investigate which communication strategies most effectively enhance patient agency, knowledge retention and self‐management. As a result of the above recommendation, findings from the TRANSFER‐I study have informed a pilot‐feasibility study which aims to improve discharge communication with a nurse‐coordinated model of care, namely the TRANSFER‐II study (registered on the Australian New Zealand Clinical Trials Registry ACTRN12624000795594 [52]), currently underway within the local health service in which this study was conducted.

Financial implications of accelerated discharge processes, driven by hospital efficiency metrics, warrant re‐evaluation. Clinicians are working hard to do their best within the current resource‐restricted environment, and future research should examine whether rapid turnover increases the risk of preventable readmissions, balancing operational efficiency with patient safety. Investigating sustainable funding models that ensure equitable access to social and domestic assistance support services must be prioritised. As hospital readmissions are costly, investing in transitional care strategies that help older adults remain safely at home is essential for future care delivery.

### 4.2 Strengths and Limitations

The age demographic forms a methodological strength, as it ensures authentic representation of the experiences of hospitalised older adults, enhancing the validity. This study employed purposive sampling to capture a range of experiences among patients and carers. While this approach supports rich, contextual insights, it does not aim for statistical representativeness. Findings should be interpreted with the context of this study in mind, and transferability of findings can be applied to comparable healthcare environments. A key limitation of the study was the requirement that all participants be English‐speaking, although English was a second language for some participants. Not all eligible participants screened were approached; if patients or their carers were engaged with members of the multidisciplinary team or unavailable and subsequently discharged, they were missed. Additionally, carers were only recruited while visiting their family members in the hospital. Participants chose the location of their interviews (either in‐person or over the phone), resulting in interviews being conducted at various points throughout the discharge process. As a result, participants could only share insights based on their experiences up to that point. Interviews often serve as a platform for participants to reflect on their experiences; as a result, some data included extends beyond transitional care but was included to represent participants' authentic experience.

## 5 Conclusion

Patient and carer experiences of hospital‐to‐home transitions highlight an urgent need for healthcare providers to enhance communication, strengthen care coordination and reduce system fragmentation. By amplifying patient and carer voices, this study reinforces the necessity of tailored discharge communication that aligns with individual needs. Integrating these perspectives into service improvements will enable healthcare systems to navigate the complexities of transitional care and drive meaningful change. The insights gained from this study have provided strong foundations for a future transitional care intervention, currently being tested in a feasibility study (TRANSFER‐II) within the same local health district.

## Author Contributions

**Kirsten Parker:** conceptualisation, methodology, data curation, investigation, validation, formal analysis, visualisation, project administration, writing – original draft. **Caleb Ferguson:** conceptualisation, methodology, formal analysis, writing – review and editing, visualisation, supervision. **Julee McDonagh:** conceptualisation, methodology, writing – review and editing, visualisation, supervision. **Richard Lindley:** conceptualisation, writing – review and editing, supervision. **Louise Hickman:** conceptualisation, methodology, validation, formal analysis, writing – review and editing, visualisation, supervision.

## Conflicts of Interest

The authors declare no conflicts of interest.

## Data Availability

As per ethical requirements, participant data will remain confidential. Anonymised data including qualitative analysis can be shared with an academic research team upon reasonable request to the corresponding author.

## Appendix A Patient Interview Guide

**Table:**

Question	
1.	Can you describe what has been the biggest change for you, since you have been admitted to the hospital?
2.	Can you tell me what information or support should be given to you to help you feel assisted and organised to return home?
3.	How and when were you told you would be leaving the hospital? Was the plan for your return home explained to you? (e.g., follow up with the GP)
4.	Can you tell me what health professionals (doctors/nurses/OT/Physio) it would be good to talk with before you return home?
5.	Can you describe to me the most important services (meals/personal care/existing services) that need to be put in place before going home so that you feel safe? Or assistance you need?
6.	If you were to have a meeting before you went home with the team of people who have looked after you in hospital, what are the things you want discussed at that meeting?
7.	We are proposing to have someone help and support you with the process of leaving the hospital—what do you think this person could do, and how can they best support you to return home?
8.	Do you have any ideas for additional support, services or other strategies that could be used to improve the service to support you leaving the hospital to return home?
9.	Thank you for taking the time today to tell me about your experiences leaving the hospital. Is there anything else you would like to add?

## Appendix B Carer Interview Guide

**Table:**

Question	
1.	Can you describe what you think has been the biggest change for ‘NAME’, since they have been admitted to the hospital?
2.	Can you tell me what information or support should be given to ‘NAME’ to help them feel assisted and organised to return home?
3.	How and when was ‘NAME’ told they would be leaving the hospital? Was the plan for their return home explained to them?
4.	Can you tell me what health professionals (doctors/nurses/OT/Physio) would be good to talk with before ‘NAME’ returns home?
5.	Can you describe to me the most important services (meals/personal care/existing services) that need to be put in place before ‘NAME’ returns home so they feel safe and supported? Or assistance they need?
6.	If you and ‘NAME’ were to have a meeting before ‘NAME’ went home with the team of people who have looked after ‘NAME’ in the hospital, what are the things you would like discussed at that meeting?
7.	We are proposing to have someone help ‘NAME’ and support them with the process of leaving the hospital—what do you think this person could do, and how can they best support ‘NAME’ to return home?
8.	Do you have any ideas for additional support, services or other strategies that could be used to improve the service to support patients leaving the hospital to return home?
9.	Thank you for taking the time today to tell me about your experience being a carer for someone who is leaving the hospital, is there anything else you would like to add?
